# Highly Scattering Hierarchical Porous Polymer Microspheres with a High-Refractive Index Inorganic Surface for a Soft-Focus Effect

**DOI:** 10.3390/polym12102418

**Published:** 2020-10-20

**Authors:** Joonsik Yoon, Ji Hyun Lee, Jun Bae Lee, Jun Hyup Lee

**Affiliations:** 1 Department of Chemical Engineering, Myongji University, Yongin 17058, Korea; sky4454278@naver.com; 2 Cosmax R&I Center, Innovation Lab, Cosmax, Seongnam 13486, Korea; jihyunlee07@cosmax.com (J.H.L.); jblee@cosmax.com (J.B.L.); 3 Department of Chemical Engineering, Soongsil University, Seoul 06978, Korea

**Keywords:** core-shell structure, diffuse reflectance, hybrid polymer particle, light scattering, soft-focus effect

## Abstract

Functional light scattering materials have received considerable attention in various fields including cosmetics and optics. However, a conventional approach based on optically active inorganic materials requires considerable synthetic effort and complicated dispersion processes for special refractive materials. Here, we report a simple and effective fabrication strategy for highly scattering hierarchical porous polymer microspheres with a high-refractive index inorganic surface that mitigates the disadvantages of inorganic materials, producing organic-inorganic hybrid particles with an excellent soft-focus effect. Hierarchical organic-inorganic hybrid particles were synthesized using the simple physical mixing of porous poly (methyl methacrylate) (PMMA) microparticles with different pore sizes and regularities as the organic core and titanium dioxide (TiO_2_) nanoparticles with different particle sizes as the inorganic shell. The polar noncovalent interactions between polar PMMA microspheres and the polar surface of TiO_2_ nanoparticles could induce the hierarchical core-shell structure of hybrid particles. The synthesized hybrid particles had increased diffuse reflectance properties of up to 160% compared with single inorganic particles. In addition, the light scattering efficiency and soft-focus effect could be increased further, depending on the size of the TiO_2_ nanoparticles and the pore characteristics of the PMMA microspheres. The proposed study can provide a facile and versatile way to improve the light scattering performance for potential cosmetics.

## 1. Introduction

Since the beginning of the industrial era, several studies have been conducted on organic-inorganic hybrid materials in various fields [1,2,3,4,5,6,7]. The combination of organic and inorganic materials improves the properties of each component while reducing the specific limitations of each material. These functional hybrid materials generally contain two or more different components, such as inorganic materials (inorganic particles, metal ions, salts, oxides, etc.) and organic materials (organic groups or molecules, organic ligands, organic polymers, etc.). They are also combined by various methods—such as self-assembly, electrostatic interaction, intermolecular interactions, and bonding in molecular structural units—to improve the synergistic effect of their functional properties. The chemical and physical bonding between organic and inorganic components can be achieved through hydrogen bonding, van der Waals bonding, ionic boding, or covalent bonding [8,9,10,11]. These organic-inorganic hybrid functional materials are widely utilized in various industries, including catalysts, drugs, optics, energy storage, environmental remediation, health, cosmetics, and packaging, and provide potential platforms for versatile applications [10,11,12,13,14,15,16,17,18,19,20,21,22,23,24,25,26]. In particular, various studies using the optical properties of organic-inorganic hybrid materials have been conducted in applications such as lenses, optical filters, optical adhesives, optical films, anti-reflective films, and cosmetics [27,28,29,30].

**Figure 1:**
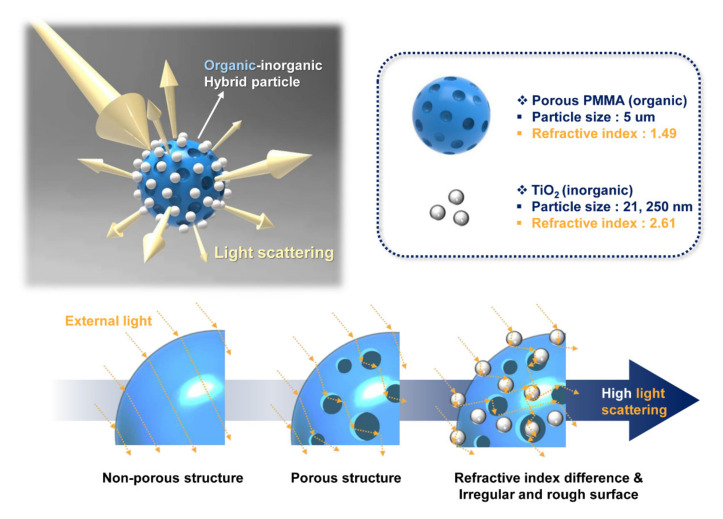
Schematic of a hierarchical organic-inorganic hybrid polymer microsphere with an excellent light scattering performance.

The soft-focus effect refers to the phenomenon in which the lens is blurred in photography. This blurring phenomenon can be achieved by soft-focus powder as a filler in various fields, and a representative example is cosmetic powder [31,32,33,34,35]. Current cosmetic foundations focus on raw materials that exhibit optical properties through high-refractive-index inorganic particles in order to provide a good coverage for three-dimensional skin defects such as wrinkles, spots, pores, and irregularities. This improves the appearance of the skin by introducing light scattering and reflection functions into an inorganic powder with a high refractive index to visually blur skin defects. Inorganic powders with such characteristics mainly include titanium dioxide, zirconium oxide, and zinc oxide. However, the high opacity of the above particles, when accumulated in three-dimensional skin, causes troubles such as pores and wrinkles, contrasts with other skin, and interferes with natural makeup [36]. A commonly used foundation is often dispersed in water or oil, and it is important that the pigment has a stable and uniform dispersion phase. However, it is difficult to stably disperse inorganic powders in water or oil, which raises concerns of aggregation on the skin [37,38,39]. In order to solve this problem, we have prepared organic-inorganic hybrid particles by combining porous organic polymer microparticles with advantages such as high oil absorption and sebum adsorption power and inorganic nanoparticles with high refractive properties (Figure 1). Organic polymer particles have a considerably lower refractive index than inorganic particles, which decreases the scattering effect of light. However, the high refractive index difference between the two materials on the bonding surface of the organic and inorganic materials is expected to greatly amplify the light scattering effect. In addition, the prepared organic-inorganic hybrid particles have an extremely irregular and rough surface owing to the porous surface of the polymer particle and inorganic nanoparticles irregularly bonded to the core particles [40,41,42,43,44]. This peculiar surface of the manufactured hybrid particles could further enhance the diffuse reflection and scattering characteristics of light.

In this study, we present a simple and effective strategy for the fabrication of hierarchical organic-inorganic hybrid polymer microparticles with a high-refractive index inorganic surface to induce a maximum light scattering performance for excellent soft-focus effect. Organic-inorganic hybrid particles were prepared using porous poly(methyl methacrylate) (PMMA) microparticles with a low refractive index (*n* = 1.49) as the organic core and titanium dioxide (TiO_2_) nanoparticles with a high refractive index (*n* = 2.61) as the inorganic shell [45,46,47]. The prepared hybrid particles enhanced the light scattering characteristics through multiple effects of the porous morphology, the refractive index difference between the organic core and inorganic shell, and the rough high-refractive index surface. Organic-inorganic hybrid particles were named “organic PMMA@inorganic TiO_2_”. The method involved inducing polar interfacial boding between two particles through physical agitation in a solvent dispersion phase in an easy, fast, and economical manner. The nanoscale morphology and particle size of the prepared organic-inorganic hybrid polymer particles were analyzed using field emission scanning electron microscopy; additionally, Fourier transform infrared spectroscopy was conducted to confirm the structure of the hybrid particles. To examine the diffuse reflectance characteristics, hybrid polymer particles were mixed with an acrylate-based resin to prepare a thin polymer film with a constant thickness. To compare the soft-focus characteristics, the prepared hybrid particles were mixed with a nitrocellulose collodion. Various optical properties according to the structure of PMMA microparticle and TiO_2_ nanoparticle were compared and discussed.

## 2. Materials and Methods

### 2.1. Materials

Titanium dioxide (TiO_2_) and poly(methyl methacrylate) (PMMA) particles were obtained from Cosmax (Seongnam, Korea). For TiO_2_, white powdery particles with average diameters of 20 nm and 250 nm were prepared using a simple sol-gel and calcination method [48]. For PMMA, round bead microparticles with different pore characteristics were synthesized through dispersion polymerization [49,50]. The characteristics of each particle, including the particle size, pore size, and pore uniformity, are shown in Table 1. Acrylate-type resin was received from TMS (Ilsan, Korea). Nitrocellulose collodion was purchased from Sigma Aldrich (Seoul, Korea).

### 2.2. Synthesis of Oranic-Inorganic Hybrid Polymer Particles

For the synthesis of organic-inorganic hybrid particles, a solution of porous PMMA and TiO_2_ nanoparticles was prepared as a first step. Porous PMMA powder (8 g) was added to 100 mL of ethanol and stirred at room temperature at 1000 rpm for 10 min. In another beaker, 8 g of TiO_2_ powder was added to 100 mL of ethanol, and then stirred at room temperature at 1000 rpm for 10 min. Thereafter, the two solutions were subjected to ultrasonic dispersion for 10 min. The solution in which the porous PMMA was dispersed was then added to the TiO_2_ dispersion solution. The resulting solution was stirred at 1000 rpm for 4 h at room temperature. In the second step, filtration was performed using filter paper (3 μm pores) to separate the uncoupled TiO_2_ nanoparticles. A vacuum pump was used for fast filtration. Since a small number of uncoupled TiO_2_ nanoparticles were found in the scanning electron microscope analysis, it is supposed that the microscale filtration process is an effective method for the removal of TiO_2_ nanoparticles. After the filtration step, the obtained hybrid particles were dried in a vacuum oven at room temperature for 24 h to remove any residual solvent.

### 2.3. Preparation of Polymer Thin Films Containing Organic-Inorganic Hybrid Particles

To measure the diffuse reflectance, polymer thin films containing hybrid particles were prepared using an acrylate-type resin containing a photoinitiator. First, 0.5 g of hybrid particles was added to 9.5 g of acrylate resin and mixed using a paste mixer of a revolution/rotation system (AR-100, Thinky, Tokyo, Japan). The mixing process was carried out at a speed of 2200/800 rpm (revolution/rotation) for 30 min. After the mixed resin was applied between two release films (polyethylene terephthalate film coated with silicon), a uniform thin film with a thickness of 150 μm was prepared using a roll-to-roll coater. The prepared film was UV-cured with 4 J cm^−2^ using a UV curing machine (KJPHT-101, KJUV, Incheon, Korea). As a result, various thin film samples including pure thin film without any particles, thin films with only organic PMMA particles, thin films with only inorganic TiO_2_ particles, and thin films with organic-inorganic hybrid particles were prepared.

### 2.4. Characterization

A Fourier transform infrared spectrometer (FT-IR, model: Agilent, Cary 660 FTIR, Santa Clara, CA, USA) was used to analyze the chemical structure of organic, inorganic, and organic-inorganic hybrid particles. The FT-IR measurement was performed by mixing the sample and KBr in the form of pellets and then subjecting them to wavelengths in the range of 4000–500 cm^−1^ (8 cm^−1^-resolution and 30 infrared scans). A field emission scanning electron microscope (FE-SEM, model: Hitachi, SU-70, Tokyo, Japan) was used to examine the morphology and size of the organic, inorganic, and organic-inorganic hybrid particles. The FE-SEM images were measured by placing the sample on carbon tape and coating it with platinum. A thermogravimetric analysis (TGA, model: TA Instruments, SDT Q-600, New Castle, DE, USA) was carried out to determine the composition of the organic-inorganic hybrid particles. Visible, near-infrared, and shortwave-infrared spectroscopy (VNIR-SWIR, model: Malvern Panalytical, ASD LabSpec 4, Malvern, UK) equipped with a contact probe was performed to examine the diffuse reflectance of thin films embedded with hybrid particles. With the black substrate as the base line, the diffuse reflectance was measured by contacting the sample with a probe that simultaneously generates incident light and detects reflected light. A goniophotometer (Murakami Color Research Laboratory, GP-5, Tokyo, Japan) was used to examine the light scattering characteristics of the hybrid particles and obtain a soft-focus factor. Each hybrid particle was mixed with nitrocellulose collodion, which is used as a makeup matrix or surgical dressing, and measured as coated on the black substrate. The scattered light was measured in the range of 0° to 180°.

## 3. Results and Discussion

### 3.1. Structural Characterization of Hierarchical Organic-Inorganic Hybrid Polymer Microspheres

**Figure 2:**
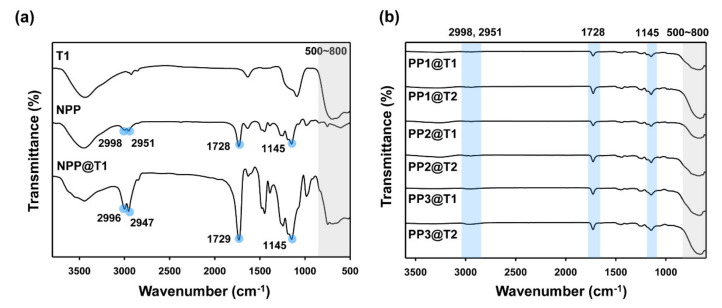
(**a**) FT-IR spectra of pure TiO_2_ (T1), pristine PMMA (NPP), and organic-inorganic hybrid particles (NPP@T1). (**b**) FT-IR spectra of other porous hybrid particles.

A FT-IR analysis was performed to analyze the chemical structure of the organic-inorganic hybrid particles synthesized by a simple mixing and drying process. Commonly, organic-inorganic hybrid particles have been prepared by a chemical bonding method using the electrostatic charge interactions between different particles [1,2,3,4,5,6,7,8,9,10,11]. However, the chemical bonding method requires a complex and long synthetic procedure. Therefore, the used physical mixing method can provide the fast and simple fabrication of hybrid particles using the polar interfacial interactions between polar PMMA microspheres and the polar surface of TiO_2_ nanoparticles. It is reported that PMMA and TiO_2_ nanoparticles can interact chemically and physically due to the presence of polar functional groups, offering an excellent compatibility between the two materials [51,52,53]. Figure 2a shows the FT-IR spectra of pure TiO_2_ nanoparticles (T1), pristine PMMA (NPP), and hybrid particles (NPP@T1). First, in the spectrum of the TiO_2_ nanoparticle, the Ti-O-Ti peak of TiO_2_ was observed broadly at 500–800 cm^−1^ [54]. In the spectrum of the PMMA polymer microparticle, C-H stretching vibration peaks were observed at 2951 and 2998 cm^−1^, and C=O carbonyl peaks were observed at 1728 and 1145 cm^−1^ [55]. Meanwhile, in the spectrum of the organic-inorganic hybrid particle (NPP@T1), the characteristic peaks of both T1 and NPP particles were observed. Therefore, it was confirmed that both the PMMA microparticles and TiO_2_ nanoparticles were incorporated into the organic-inorganic hybrid polymer microparticles. Figure 2b shows the FT-IR spectra of the prepared organic-inorganic hybrid particles. Similar spectral features were obtained for other hybrid particles, indicating the successful preparation of organic-inorganic hybrid particles.

**Figure 3:**
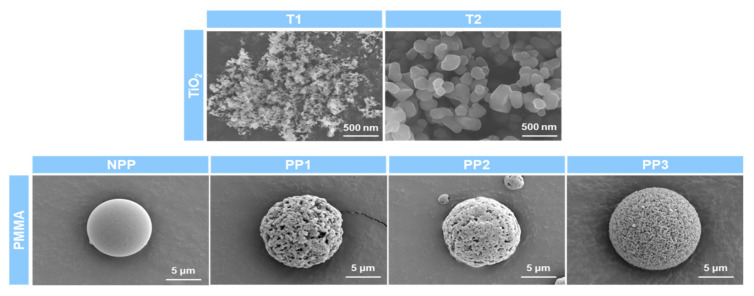
FE-SEM images of pure TiO_2_ nanoparticles and pristine PMMA microparticles.

**Figure 4:**
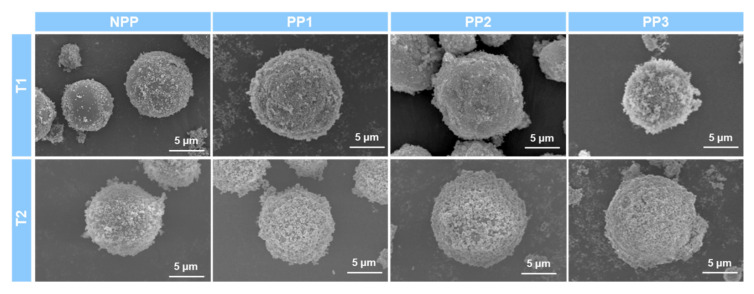
FE-SEM images of hierarchical organic-inorganic hybrid particles.

Next, an FE-SEM analysis was performed to confirm the size and morphology of the organic-inorganic hybrid particles. Figure 3 shows FE-SEM images of pure TiO_2_ nanoparticles and pristine PMMA microparticles. The T1 and T2 nanoparticles exhibited angular shapes of about 20 and 250 nm, respectively. In the case of pristine PMMA, spherical particles with average diameters of about 10 μm were observed. While non-porous NPP has a smooth surface without pores, the three porous PMMA particles have a large number of pores with different sizes and regularities, as summarized in Table 1. Notably, PP1 and PP3 showed uniform pore diameters of about 400 and 200 nm, respectively, and PP2 exhibited a nonuniform pore size of about 300 nm. Figure 4 shows FE-SEM images of the prepared organic-inorganic hybrid particles. All the hybrid particles maintained the spherical morphologies of pristine PMMA microparticles, however their surface morphologies were changed to irregular and rough surfaces with a great number of TiO_2_ protrusions, suggesting the successful binding between PMMA microparticles and TiO_2_ nanoparticles. The polar interfacial interactions between polar PMMA microspheres and the polar surface of TiO_2_ nanoparticles could lead to the hierarchical core-shell structure of hybrid particles. These irregular and rough surfaces of the hybrid core-shell particles with high-refractive index TiO_2_ protrusions are expected to enhance the light scattering and diffuse reflection characteristics.

**Figure 5:**
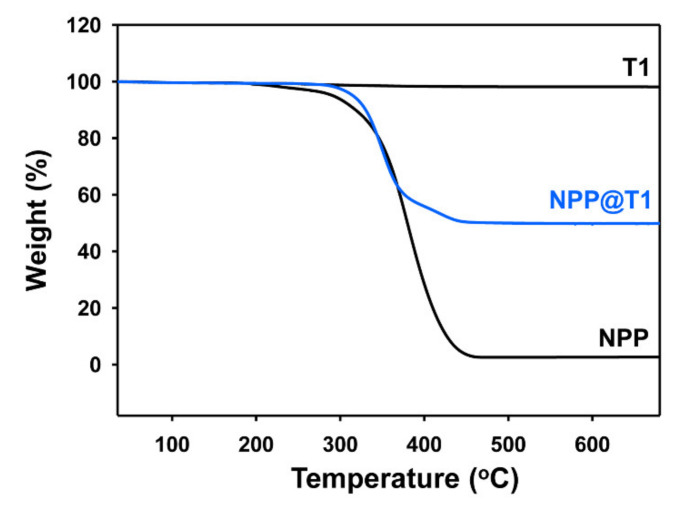
TGA thermograms of pure TiO_2_ (T1), pristine PMMA (NPP), and hybrid particles (NPP@T1).

To quantitatively determine the composition of the organic-inorganic hybrid particles, a thermogravimetric analysis was performed. Figure 5 shows the TGA thermograms of pure TiO_2_ (T1), pristine PMMA (NPP), and organic-inorganic hybrid particles (NPP@T1). While the pure TiO_2_ particles maintained their initial weight even at 600 °C, a complete degradation at 500 °C was observed for pristine PMMA. In addition, a final weight loss of about 49% was detected for the NPP@T1 hybrid particle, indicating that the organic-inorganic hybrid particle prepared with a mixing ratio of 1:1 retains the exact chemical composition of TiO_2_:PMMA = 1:1.

### 3.2. Optical Properties of Hierarchical Organic-Inorganic Hybrid Polymer Microspheres

**Figure 6:**
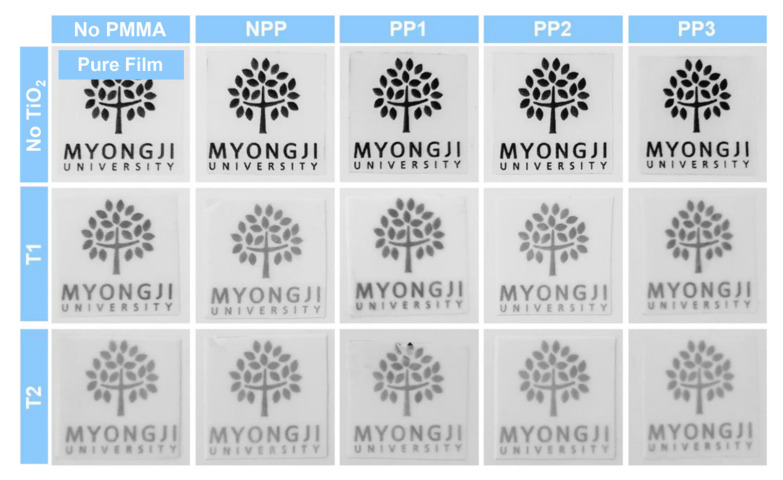
Photographs of the pure thin film and thin films with only PMMA, only TiO_2_, and hybrid particles.

Figure 6 shows photographs of the thin polymer films embedded with organic-inorganic hybrid particles. In order to visually compare the actual appearance and transparency of the films, the manufactured films were cut into 3 × 3 cm^2^ sizes, respectively, and placed on paper printed with the logo. The first row showed pure thin films without any particles and thin films with only PMMA microparticles. These thin films exhibited transparent logo images due to the good compatibility and similar refractive indices between the PMMA microparticles and the acrylate-based matrix resin. The second and third rows displayed the thin films embedded with only TiO_2_ nanoparticles and hybrid particles, and opaque and blurry images were obtained for all samples compared to those of the pure and PMMA-embedded thin films. This result is ascribed to the light scattering effect of high-refractive index TiO_2_ nanoparticles in the low-refractive index acrylate-based matrix. Notably, the thin films with large-sized TiO_2_ nanoparticles (T2) showed much blurrier images than those with small TiO_2_ (T1) [56]. Based on these results, thin films embedded with organic-inorganic hybrid particles are expected to have good light scattering characteristics comparable to those with only inorganic TiO_2_ nanoparticles.

**Figure 7:**
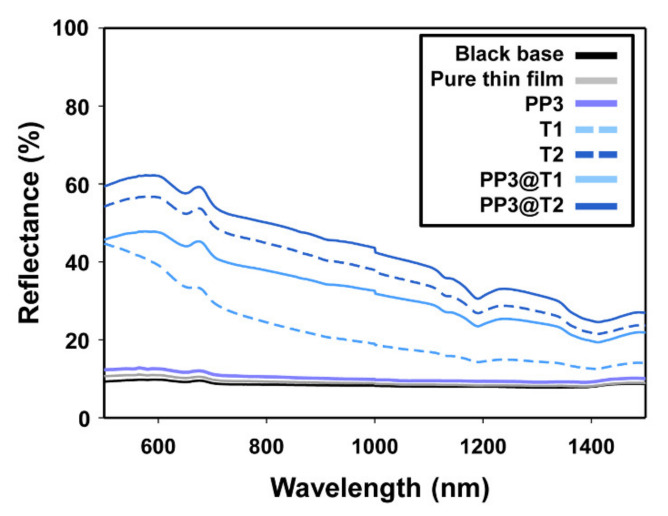
Diffuse reflectance spectra of the black substrate, pure thin film, and thin films with PP3, T1, T2, PP3@T1, and PP3@T2 particles.

To examine the difference in the light scattering performance, the diffuse reflectance spectra of the pure film and thin films with only PMMA, only TiO_2_ nanoparticles, and hybrid particles were measured and their diffuse reflectances at 600 nm were compared [57]. A diffuse reflectance analysis on the thin films was performed using contact reflectance measuring equipment. The prepared thin films were placed on a black substrate, and incident light generated from a contact probe was absorbed or reflected by the thin films embedded with light scattering particles. Figure 7 shows the diffuse reflectance spectra of the pure film and thin films with only PMMA (PP3), only TiO_2_ nanoparticles (T1 and T2), and hybrid particles (PP3@T1 and PP3@T2). Pure thin film without any particles and thin films with only PP3 polymer showed low diffuse reflectances of approximately 10% and 13% at a wavelength of 600 nm due to the low refractive indices of PMMA microparticles and acrylate-based matrix, which is in accordance with the visual analysis results. On the contrary, the thin films embedded with only T1 or T2 nanoparticles exhibited high diffuse reflectances of approximately 39% and 57%, respectively, owing to the high refractive index of TiO_2_ nanoparticles [58]. In addition, the introduction of organic-inorganic hybrid particles into the thin films led to the improved diffuse reflectances of 48% and 62% for PP3@T1 and PP3@T2, respectively. These results suggest that the rough surface of the hybrid porous PMMA microsphere with high-refractive index TiO_2_ protrusions induces enhanced light scattering characteristics for organic-inorganic hybrid particles. Moreover, hybrid particles with large TiO_2_ nanoparticles (PP3@T2) showed a higher diffuse reflectance than those with small TiO_2_ (PP3@T1) due to the rougher surface structure. Table 2 and Table 3 summarize the diffuse reflectance characteristics of all the thin films at 600 nm. The organic-inorganic hybrid structure including the porous PMMA microsphere as the core and the large-sized TiO_2_ nanoparticles as the shell could maximize the light scattering performance. As a consequence, the thin film embedded with the PP3@T2 hybrid particle showed an about 620% higher diffuse reflectance than the pure thin film, which was 488% higher than the thin film with only NPP polymer, 160% higher than the thin film with only inorganic T1 nanoparticles, and 110% higher than the thin film with only T2 nanoparticles.

### 3.3. Soft-Focus Properties of Hierarchical Organic-Inorganic Hybrid Polymer Microspheres

**Figure 8:**
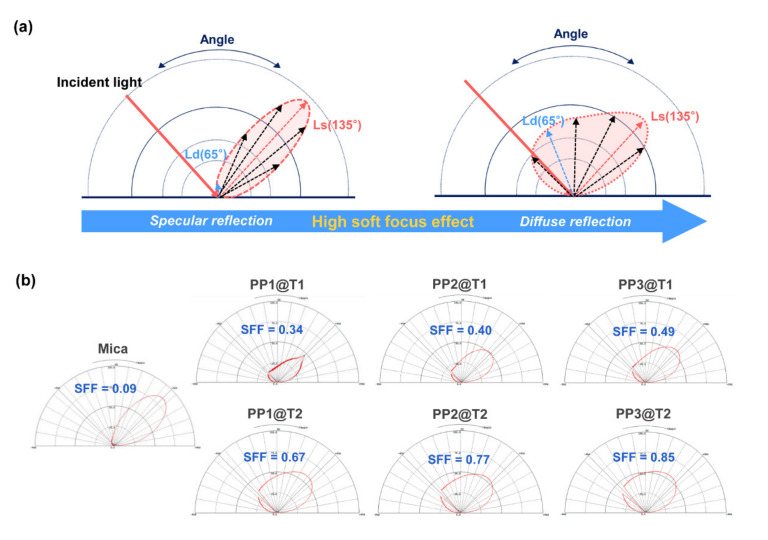
(**a**) Schematic of the goniophotometer analysis. (**b**) Light intensity distribution curves of the mica and hybrid particles.

To compare the soft-focus characteristics of the hybrid particles for potential cosmetic applications, light intensity distribution curves were measured using a goniophotometer. A schematic diagram of the goniophotometer analysis is shown in Figure 8a. Incident light from 45° was applied to the sample, and the reflected light in the range of 0°–180° was measured by the detector. The light intensities for specular reflection at 135° and diffuse reflection at 65° were named Ls and Ld, respectively. Especially, Ld can represent the cover effect of hybrid particles on skin defects due to the diffuse light scattering and resulting opacity. The soft-focus factor (SFF) for the quantitative light scattering performance is defined as in Equation (1) [59].

(1) $$\mathrm{Soft}\;\mathrm{focus}\;\mathrm{factor}\;\left(\mathrm{SFF}\right)\;=\frac{Ld}{Ls}$$

An SFF value of more than 0.5 indicates a good soft-focus performance, and the maximum soft-focus effect of particles can be achieved with an extremely high SFF value close to 1.0 [60]. The excellent light scattering performance of the hybrid particles can lead to high SFF values due to the increased diffuse reflection. Figure 8b shows the light intensity distribution curves of mica as a reference material for cosmetic application and hybrid particles. While the mica particles exhibited extremely low diffuse reflectance due to their flat and smooth surface, an enhanced diffuse reflection was observed for the organic-inorganic hybrid particles. In addition, the hybrid particles with large TiO_2_ nanoparticles showed excellent soft-focus effects compared to those with small TiO_2_, which is quite in accord with the diffuse reflectance results.

**Figure 9:**
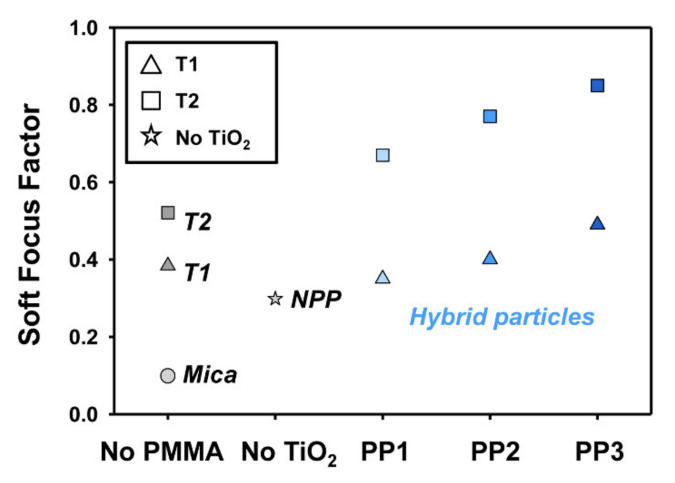
Soft-focus factor values of mica, T1, T2, NPP, and hybrid particles.

Figure 9 shows the SFF values of mica, pure TiO_2_ (T1 and T2), pristine PMMA (NPP), and hybrid particles. The organic-inorganic hybrid porous particles showed remarkably high SFF values compared to those of the conventional mica particles. The large TiO_2_ nanoparticles and irregular or small pore size porous PMMA particles resulted in high SFF values and excellent soft-focus effects. Basically, the refractive index difference between the PMMA core and the inorganic TiO_2_ shell induces good light scattering properties for hybrid particles and, furthermore, the irregular and rough surface leads to an additional improvement in the light scattering performance. Consequently, the PP3@T2 hybrid particle showed the highest SFF value of 0.85, which is about 944% higher than that of mica particles, 285% higher than that of pristine PMMA polymer, and 163% higher than that of pure T2 nanoparticles.

## 4. Conclusions

In this study, we fabricated highly scattering hierarchical porous polymer microspheres with a high-refractive index inorganic surface for an excellent soft-focus effect. Nano-sized TiO_2_ and micro-sized porous PMMA with different pore sizes and regularities were used as organic and inorganic materials for the synthesis of hierarchical hybrid particles. The thin film embedded with organic-inorganic hybrid particles showed 488% or 160% higher diffuse reflectance characteristics than those with only PMMA or TiO_2_ particles. The synergistic effects of the porous morphology, the refractive index difference between the organic core and inorganic shell, and the rough high-refractive index surface can provide high light scattering properties for hybrid particles. In addition, the PP3@T2 hybrid particle comprising porous PMMA microspheres with the smallest pore size and large TiO_2_ nanoparticles exhibited the highest SFF value among the prepared hybrid particles. The light scattering characteristics could be greatly improved by controlling the pore characteristics of the organic PMMA particles and the size of the inorganic TiO_2_ nanomaterial. Based on these results, it was confirmed that the pore characteristics of the polymer microsphere and the size of high-refractive index inorganic material are important factors for the design of highly scattering organic-inorganic hybrid particles. This study can provide an effective and versatile approach for achieving highly light-scattering materials with excellent soft-focus effects for potential cosmetic applications including skin coverage.

## Figures and Tables

**Table 1:** Characteristics of PMMA microparticles and TiO_2_ nanoparticles.

No.	Code	Material	Average Particle Size	Average Pore Size	Pore Uniformity
1	NPP	PMMA	9 μm	non-porous	-
2	PP1	PMMA	12 μm	400 nm	uniform
3	PP2	PMMA	11 μm	300 nm	nonuniform
4	PP3	PMMA	13 μm	200 nm	uniform
5	T1	TiO_2_	20 nm	non-porous	-
6	T2	TiO_2_	250 nm	non-porous	-

**Table 2:** Diffuse reflectance of the pure thin film and thin films with only PMMA or TiO_2_ particles at 600 nm.

Materials	Pure Film	NPP	PP1	PP2	PP3	T1	T2
**Reflectance (%)**	10.0 ± 0.4	12.7 ± 0.3	12.7 ± 0.1	12.6 ± 0.3	12.7 ± 0.2	39.1 ± 0.6	56.6 ± 0.8

**Table 3:** Diffuse reflectance of the thin films with organic-inorganic hybrid particles at 600 nm.

Materials	NPP@T1	NPP@T2	PP1@T1	PP1@T2	PP2@T1	PP2@T2	PP3@T1	PP3@T2
**Reflectance (%)**	42.2 ± 0.7	55.8 ± 0.2	46.6 ± 0.5	59.1 ± 0.8	48.0 ± 0.3	58.4 ± 0.7	47.6 ± 0.4	62.0 ± 1.1

## References

[1] Sanchez C., Lebeau B. (2001). Design and Properties of Hybrid Organic-inorganic Nanocomposites for Photonics. MRS Bull..

[2] Chujo Y., Saegusa T. (1992). Organic Polymer Hybrids with Silica Gel Formed by Means of the Sol-gel Method. Adv. Polym. Sci..

[3] Ananikov V.P. (2019). Organic-Inorganic Hybrid Nanomaterials. Nanomaterials.

[4] Pardo R., Zayat M., Levy D. (2011). Photochromic Organic-inorganic Hybrid Materials. Chem. Soc. Rev..

[5] Sanchez C., Julián B., Belleville P., Popall M. (2005). Applications of Hybrid Organic-inorganic Nanocomposites. J. Mater. Chem..

[6] Mei S., Pan M., Wang J., Zhang X., Song S., Li C., Liu G. (2020). Self-Assembly of Strawberry-Like Organic-inorganic Hybrid Particle Clusters with Directionally Distributed Bimetal and Facile Transformation of the Core and Corona. Polym. Chem..

[7] Yu Y., Chen C., Chen W. (2003). Synthesis and Characterization of Organic-inorganic Hybrid Thin Films from Poly (Acrylic) and Monodispersed Colloidal Silica. Polymer.

[8] Ogoshi T., Itoh H., Kim K., Chujo Y. (2002). Synthesis of Organic-Inorganic Polymer Hybrids having Interpenetrating Polymer Network Structure by Formation of Ruthenium-Bipyridyl Complex. Macromolecules.

[9] Saveleva M.S., Eftekhari K., Abalymov A., Douglas T.E., Volodkin D., Parakhonskiy B.V., Skirtach A.G. (2019). Hierarchy of Hybrid materials—The Place of Inorganics-in-Organics in it, Their Composition and Applications. Front. Chem..

[10] Kickelbick G. (2003). Concepts for the Incorporation of Inorganic Building Blocks into Organic Polymers on a Nanoscale. Prog. Polym. Sci..

[11] Judeinstein P., Sanchez C. (1996). Hybrid organic-inorganic Materials: A Land of Multidisciplinarity. J. Mater. Chem..

[12] Chen W., Lee S., Lee L., Lin J. (1999). Synthesis and Characterization of Trialkoxysilane-Capped Poly (Methyl Methacrylate)-titania Hybrid Optical Thin Films. J. Mater. Chem..

[13] Huynh W.U., Dittmer J.J., Alivisatos A.P. (2002). Hybrid Nanorod-Polymer Solar Cells. Science.

[14] Biteau J., Chaput F., Lahlil K., Boilot J., Tsivgoulis G.M., Lehn J., Darracq B., Marois C., Lévy Y. (1998). Large and Stable Refractive Index Change in Photochromic Hybrid Materials. Chem. Mat..

[15] Wakiya T., Morisaki T., Ishibashi N., Nishimura S., Takafuji M., Nagaoka S., Yamada Y., Nozato S., Ihara H. (2011). Preparation of multilayered organic–inorganic hybrid core–shell particles by stepwise surface formation. Mater. Lett..

[16] Huang W., Ho S., Kwei T., Okamoto Y. (2002). Photoluminescence Behavior of Poly(Quinoline)s in Silica Glasses Via the sol-gel Process. Appl. Phys. Lett..

[17] Lee T., Park O.O., Yoon J., Kim J. (2001). Polymer-layered Silicate Nanocomposite light-emitting Devices. Adv. Mater..

[18] Xu C., Eldada L., Wu C., Norwood R.A., Shacklette L.W., Yardley J.T., Wei Y. (1996). Photoimageable, Low Shrinkage Organic− Inorganic Hybrid Materials for Practical Multimode Channel Waveguides. Chem. Mat..

[19] Kagan C.R., Mitzi D.B., Dimitrakopoulos C.D. (1999). Organic-Inorganic Hybrid Materials as Semiconducting Channels in Thin-Film Field-Effect Transistors. Science.

[20] Coltrain B.K., Landry C.J., O’Reilly J.M., Chamberlain A.M., Rakes G.A., Sedita J.S., Kelts L.W., Landry M.R., Long V.K. (1993). Role of Trialkoxysilane Functionalization in the Preparation of Organic-Inorganic Composites. Chem. Mat..

[21] Yoshida M., Prasad P.N. (1996). Sol− Gel-Processed SiO_2_/TiO_2_/poly (Vinylpyrrolidone) Composite Materials for Optical Waveguides. Chem. Mat..

[22] Wang B., Wilkes G., Hedrick J., Liptak S., McGrath J. (1991). New High-Refractive-Index organic/inorganic Hybrid Materials from Sol-Gel Processing. Macromolecules.

[23] Katagiri K., Koumoto K., Iseya S., Sakai M., Matsuda A., Caruso F. (2009). Tunable UV-Responsive Organic− Inorganic Hybrid Capsules. Chem. Mat..

[24] Guo R., Du X., Zhang R., Deng L., Dong A., Zhang J. (2011). Bioadhesive Film Formed from a Novel organic-inorganic Hybrid Gel for Transdermal Drug Delivery System. Eur. J. Pharm. Biopharm..

[25] Tang J., Wang C., Wang Y., Sun J., Yang B. (2001). An Oligo-Phenylenevinylene Derivative Encapsulatedin sol-gel Silica Matrix. J. Mater. Chem..

[26] Lee L., Chen W. (2001). High-Refractive-Index Thin Films Prepared from Trialkoxysilane-Capped Poly (Methyl Methacrylate)− Titania Materials. Chem. Mat..

[27] Draxl C., Nabok D., Hannewald K. (2014). Organic/inorganic Hybrid Materials: Challenges for Ab Initio Methodology. Acc. Chem. Res..

[28] Chang C., Chen W. (2001). High-refractive-index Thin Films Prepared from Aminoalkoxysilane-capped Pyromellitic dianhydride-titania Hybrid Materials. J. Polym. Sci. A Polym. Chem..

[29] Lü C., Guan C., Liu Y., Cheng Y., Yang B. (2005). PbS/polymer Nanocomposite Optical Materials with High Refractive Index. Chem. Mat..

[30] Mimura S., Naito H., Kanemitsu Y., Matsukawa K., Inoue H. (2000). Optical Properties of organic-inorganic Hybrid Thin Films Containing Polysilane Segments Prepared from polysilane-methacrylate Copolymers. J. Organomet. Chem..

[31] Su C., Tang H., Zhu G., Li C., Lin C. (2014). The Optical Properties and Sunscreen Application of Spherical h-BN-TiO_2_/mica Composite Powder. Ceram. Int..

[32] Okamoto T., Kumagawa T., Motoda M., Igarashi T., Nakao K. (2013). Monte Carlo Simulation of Light Reflection from Cosmetic Powder Particles Near the Human Skin Surface. J. Biomed. Opt..

[33] Igarashi T., Nishino K., Nayar S.K. (2007). The Appearance of Human Skin: A Survey. Found. Trends Comput. Graph. Vis..

[34] Welcomme E., Walter P., Van Elslande E., Tsoucaris G. (2006). Investigation of White Pigments used as make-Up during the Greco-Roman Period. Appl. Phys. A.

[35] Anderson R.R., Parrish J.A. (1981). The Optics of Human Skin. J. Investig. Dermatol..

[36] Dréno B., Alexis A., Chuberre B., Marinovich M. (2019). Safety of Titanium Dioxide Nanoparticles in Cosmetics. J. Eur. Acad. Dermatol. Venereol..

[37] Almusallam A.S., Abdulraheem Y.M., Shahat M., Korah P. (2012). Aggregation Behavior of Titanium Dioxide Nanoparticles in Aqueous Environments. J. Dispers. Sci. Technol..

[38] Okuda-Shimazaki J., Takaku S., Kanehira K., Sonezaki S., Taniguchi A. (2010). Effects of Titanium Dioxide Nanoparticle Aggregate Size on Gene Expression. Int. J. Mol. Sci..

[39] Domingos R.F., Peyrot C., Wilkinson K.J. (2010). Aggregation of Titanium Dioxide Nanoparticles: Role of Calcium and Phosphate. Environ. Chem..

[40] Bishop M.T., Langley K.H., Karasz F.E. (1989). Dynamic Light-Scattering Studies of Polymer Diffusion in Porous Materials: Linear Polystyrene in Porous Glass. Macromolecules.

[41] Bishop M., Langley K., Karasz F. (1986). Diffusion of a Flexible Polymer in a Random Porous Material. Phys. Rev. Lett..

[42] Elson J. (1984). Theory of Light Scattering from a Rough Surface with an Inhomogeneous Dielectric Permittivity. Phys. Rev. B.

[43] Knotts M., Michel T., O’Donnell K. (1993). Comparisons of Theory and Experiment in Light Scattering from a Randomly Rough Surface. J. Opt. Soc. Am. A.

[44] Marvin A., Toigo F., Celli V. (1975). Light Scattering from Rough Surfaces: General Incidence Angle and Polarization. Phys. Rev. B.

[45] Prajzler V., Klapuch J., Lyutakov O., Hüttel I., Špirková J., Nekvindová P., Jeřábek V. (2011). Design, Fabrication and Properties of Rib Poly (Methylmethacrylimide) Optical Waveguides. Radioengineering.

[46] Beadie G., Brindza M., Flynn R.A., Rosenberg A., Shirk J.S. (2015). Refractive Index Measurements of Poly (methyl methacrylate)(PMMA) from 0.4–1.6 μm. Appl. Opt..

[47] Paulsen M., Neustock L.T., Jahns S., Adam J., Gerken M. (2017). Simulation Methods for Multiperiodic and Aperiodic Nanostructured Dielectric Waveguides. Opt. Quant. Electron..

[48] Haque F.Z., Nandanwar R., Singh P. (2017). Evaluating Photodegradation Properties of Anatase and Rutile TiO_2_ Nanoparticles for Organic Compounds. Optik.

[49] Chiu W., Chen Y., Tsai P., Wu J. (2016). Preparation and Characterization of Poly(Methyl Methacrylate) Microbeads by Dispersion Polymerization: Effects of the Medium Composition, Monomer Concentration, Thermal, and Optical Properties. Polym. Plast. Tech. Eng..

[50] Kim D., Lee D.Y., Lee K., Choe S. (2009). Effect of Crosslinking Agents on the Morphology of Polymer Particles Produced by One-Step Seeded Polymerization. Macromol. Res..

[51] Chatterjee A. (2010). Properties Improvement of PMMA Using Nano TiO_2_. J. Appl. Polym. Sci..

[52] Hafizah N.N., Mamat M.H., Abidin M.H., Said C.M.S., Rusop M. (2014). Bonding and Mechanical Properties of PMMA/TiO_2_ Nanocomposites. Adv. Mater. Res..

[53] Gad M.M., Abualsaud R. (2019). Behavior of PMMA Denture Base Materials Containing Titanium Dioxide Nanoparticles: A Literature Review. Int. J. Biomater..

[54] Nasikhudin Ismaya E.P., Diantoro M., Kusumaatmaja A., Triyana K. (2017). Preparation of PVA/TiO_2_ Composites Nanofibers by using Electrospinning Method for Photocatalytic Degradation. IOP Conf. Ser. Mater. Sci. Eng..

[55] Mohan K., Dolui S., Nath B.C., Bora A., Sharma S., Dolui S.K. (2017). A Highly Stable and Efficient Quasi Solid State Dye Sensitized Solar Cell Based on Polymethyl Methacrylate (PMMA)/Carbon Black (CB) Polymer Gel Electrolyte with Improved Open Circuit Voltage. Electrochim. Acta.

[56] Tsai L., Yang P.N., Shih Y., Lin K., Wu C., Lin H.Y. (2017). Size-Dependent Multiple-Scattering Effects of Mesoporous TiO_2_ Beads Distinguished by Optical Coherence Tomography. IEEE Photonics J..

[57] Son I., Lee J.H. (2020). Highly Transparent and Wide Viewing Optical Films Using Embedded Hierarchical Double-Shell Layered Nanoparticles with Gradient Refractive Index Surface. ACS Appl. Mater. Interfaces.

[58] Osiris W., Mohamed S., El-Zaher N. (2013). Macrostructure and Optical Study of PMMA/TiO_2_ Nanopartcles Composites. Nano Sci. Nano Technol. Indian J..

[59] Choi Y., Choi J., Kim H. (2019). Shape Control of Silica-Polymethylsilsesquioxane (PMSQ) Composites by Varying Ratios of Precursors. J. Soc. Cosmet. Korea.

[60] Becker M., Schmidt C., Hochstein V., Petsitis X. (2012). A Novel Method to Measure and Pre-select Functional Filler Pigments. Cosmet. Toilet..

